# Comprehensive Environmental Assessment Index of Ecological Footprint

**DOI:** 10.1007/s00267-022-01747-z

**Published:** 2022-11-18

**Authors:** Mohsen Khezri, Mahnaz Mamghaderi, Somayeh Razzaghi, Almas Heshmati

**Affiliations:** 1 Department of Business and Management, School of Management and Economics, University of Kurdistan Hewlêr, 30 Meter Avenue, Erbil, Kurdistan Region Iraq; 2 Student of Industrial Engineering, Iran University of Science and Technology, Tehran, Iran; 3 Faculty of Economics and Social Sciences, Bu-Ali Sina University, Hamedan, Iran; 4 Jönköping International Business School, Room B5017, Gjuterigatan 5, SE-551 11, Jönköping, Sweden

**Keywords:** Environmental Performance, Ecological footprint, KPI, DEA, OECD Countries, F64, O44, O47, Q56, Q57

## Abstract

This paper aims to contribute to the growing body of research literature on assessing environmental efficiency by introducing a new key performance indicator (KPIs) in more complete and dependable aspects of ecological footprint indices. For this purpose, the DEA model considering three inputs (energy consumption, labor force, and capital stock), one desirable output (GDP), and different undesirable outputs (CO_2_ emissions, ecological footprint indicators) are applied to 27 OECD countries from 2000 to 2017. According to the results, Norway, Luxemburg, and United Kingdom are the most environmentally efficient countries in terms of environmental efficiency and ecological footprint efficiency. On the other hand, the lowest environmental and ecological footprint efficiencies were in countries like Lithuania, Slovak, Czech, Estonia, and the USA. In addition, these nations fare poorly regarding their carbon footprint and farmland efficiency. In further detail, Lithuania, South Korea, Portugal, and Spain have a critical status in fishing ground efficiency, while the forest area efficiency is very acute in Estonia, Latvia, Lithuania, and Czech.

## Introduction

According to the Global Risks Report 2022 (McLennan and Group 2022), environmental degradation is one of the five most significant threats to the global economy in the following decades. Continued environmental deterioration can destroy all environmental elements, including natural resources, ecosystems, biodiversity, and habitats. It has grave consequences for humanity, including cultural destruction, famine, forced migration, sickness, and even war. The World Health Organization (WHO) states that worsening environmental conditions account for over 24 percent of all predicted worldwide fatalities. By 2030, their direct annual health costs are projected to range between $2 and $4 billion. It is crucial to restrict such damaging impacts and increase environmental efficiency to avert ecological and social repercussions of environmental degradation.

Economic growth is one of the most significant contributors to environmental degradation (M. Usman et al. 2020), and countries with high economic activity have more detrimental environmental effects. According to the National Footprint and Biocapacity Accounts (NFAs) reports for 2020, more than 85 percent of human lives will be ecologically insufficient. The Earth has been experiencing an overshoot, in which civilization consumes more resources and produces more waste (Wackernagel et al. 2019). In this context, the OECD countries shared more than 60 percent of the world GDP and exports in 2020, and they have material-intensive economies releasing the most carbon dioxide (WDI, 2020). Consequently, they recorded the shrinking share of agricultural lands in Western Europe countries, a significant percentage of the world’s pesticide sales (37% of all pesticides), and an accelerated declining rate of the farmland bird index. These are raised by the extent of crop fields without trees, increased trends in agricultural ammonia and greenhouse gas emissions (yearly 0.2%), increased nitrogen fertilizer application rates, expanded irrigation areas, increased reliance on underground water rather than surface water, and increased nitrogen balances in several OECD countries. These are the destructive outcomes of economic growth in OECD countries that exert intense pressure on the environment. These reports show the importance of empirical studies monitoring the environmental performance in OECD countries, considering all aspects of environmental devastation arising from economic activities. Therefore, we concentrate our empirical inquiry on evaluating the environmental efficiency of OECD countries, although our innovative technology is also applicable worldwide.

Data Envelopment Analysis (DEA) has been extensively used in the scientific literature to examine environmental performance. It offers a suitable way for calculating relative efficiency scores and performance indicators using numerous inputs and outputs. Any economic production activity is a joint-production process that uses energy resources, labor force, and capital stock to create both desirable outputs (such as GDP) and undesirable outputs (such as the emission of pollutants) from an economic perspective. Therefore, a bulk of studies are considering environmental performance in different regions and applying various DEA methodologies. Matsumoto et al. (2020), Woo et al. (2015), Xie et al. (2014), Zhou et al. (2006), Zhou et al. (2007), and Färe et al. (2004), among others, have investigated the environmental performance of OECD countries and underline only air pollution (CO_2_, SO_2_, and NO_2_ emission) as the undesirable output of economic activities. However, energy consumption alongside labor force and capital stock in the production process doesn’t only cause air pollution, and it leaves other footprints on other aspects of the environment, such as water pollution, soil pollution, and Land devastation (Miliband 2016).

Consequently, a more comprehensive variable is necessary as an undesirable outcome to quantify the true impacts of economic operations on environmental quality and to calculate correct environmental efficiency ratings. In this concept, the ecological footprint measures the human impact on the environment. It demonstrates the demand for ecological assets and natural capital, such as grazing land, fishing grounds, cropland, built-up land, forest areas, and carbon demand on land, in the process of producing goods and services to support people and the economy. It assists people in assessing and controlling the use of resources throughout the economy and illustrates the size of human demand compared to what the world can replenish. Although the ecological footprint also has some weaknesses, such as not considering technological change and resources beneath the earth’s surface (Subekti and Suroso 2018), it is much more complete than the previously used indices. Therefore, using the ecological footprint as a negative output might be a suitable alternative for assessing environmental performance in nations since it thoroughly demonstrates the destructive consequences of economic activity on the environment.

In addition, in the context of the DEA, Key Performance Indicators (KPIs) are created based on environmental efficiency scores, with carbon efficiency and energy efficiency being two of the most often used KPIs in the related literature (Choi et al. 2012; Iftikhar et al. 2018; Park et al. 2016). Accordingly, we propose ecological footprint efficiency as a substitute for carbon efficiency. In addition, five additional KPIs are constructed based on the various ecological footprint categories to assess carbon footprint efficiency, cropland efficiency, fishing grounds efficiency, forest area efficiency, and grazing land efficiency performances. Such comprehensive performance indicators set objectives for attaining higher environmental efficiency, assess the present state of each component of the ecological footprint, and analyze the process of reaching environmental objectives by country in more depth. These indicators highlight environmental protection, the reduction of environmental pressures on human health, the improvement of ecosystem health, and the management of natural resources properly.

Therefore, this research contributes to the existing knowledge about measuring environmental performance in two critical areas. This article is the first effort to analyze environmental performance using ecological footprint and its specific categories as complete measurements of undesirable outcomes in the framework of the DEA methodology. Second, we offer new KPIs to thoroughly assess the ecological footprint’s efficiency and the performance of its components. These metrics will allow a more accurate examination of each nation’s ecological performance. To our knowledge, such a comprehensive assessment of OECD nations’ environmental performance has been conducted for the first time.

This paper is organized into five sections. After presenting the introduction in the first section, the related literature has been reviewed in section Literature Review. Methodology and results have been provided in sections Research Methodology and Results and Discussion, and finally, section Conclusion concludes the paper.

## Literature Review

### Environmental Efficiency and Ecological Footprint

In the 1980s, environmental sustainability concerns were emphasized, and concurrently with introducing this value, the manufacturing process’s efficiency has garnered increasing attention. Färe et al. (1989) argued that inputs in the production process generate both desired (as measured by GDP) and undesired output (as pollution). Later, Hu and Wang (2006) proposed the TFEE concept and argued that a proper combination of energy with other production factors such as labor and capital might produce output. Therefore, while analyzing the environmental efficiency of a process, we must consider both desirable and undesired inputs and outputs. Energy consumption is inseparable from production and economic activity (Kraft and Kraft 1976), establishing a connection between economic growth and energy consumption (Tiba and Omri 2017).

Given that fossil fuels play the most significant role in delivering the required energy for the industrial process, the destruction of the environment and the release of vast quantities of carbon, wastewater, and waste gas as undesired outputs are inevitable (Wu et al. 2016; Xu et al. 2020). In addition to natural resources, economic activities utilize mineral resources, water, forest, and land resources (Y. Li et al. 2019). Rapid economic expansion stimulates the use of these natural resources in a way that jeopardizes ecological sustainability (Todaro and Smith 2011). Environmental contamination may be a consequence of the overuse of natural resources.

Numerous indexes and methods are raised to scout sustainable development status worldwide. Environmental efficiency is one of the most important indexes in determining sustainable development (Song et al. 2013). Many researchers have focused on limiting energy consumption and carbon dioxide emissions to achieve a high level of environmental quality. In traditional industrialization and economic development, more attention has been paid to the environment to prevent environmental degradation. In addition, Wackernagel and Rees (1997) and Wackernagel et al. (2019) defined Ecological Footprint (EF) as an indicator of sustainability and measured environmental quality that shows the bio-capacity necessary for an economic system to sustain economic activity (Sbia et al. 2014). Then, there is a lengthy debate regarding the selection of ecological footprint as an indicator of environmental sustainability. (Wiedmann and Barrett 2010) discussed EF as a headline indicator for sustainability decision-making. (Kissinger and Haim 2008) used EF indicator to compare the ecological sustainability of Israel as a case study and declared that EF provides a valuable indicator of human ecological sustainability. Templet 2000 compared ecological footprint to the net primary productivity (NPP) across 95 countries and concluded that EF method is useful because it disaggregates the footprint into its component and can provide a reasonable level of detail for the policy maker.. EF reflects human dependence on the ecological system, so it can serve as a viable indicator for determining environmental quality (Bani Yami et al. 2021; Galli 2015). In recent years, the ecological footprint developed by Wackernagel and Rees (1997) has been recognized as the measure of environmental deterioration since it considers agriculture, fishing grounds, grazing land, carbon footprint, forestland, and developed land. In addition to CO_2_ emissions, the monitoring of air, water, and land quality has become vital due to the undeniable influence of economic activities on these ecological indices (Wackernage and Rees 1997).

### Economic activities- environmental performance

Theoretically, several drivers of environmental deterioration have been identified by scientists, including human activity, energy consumption, industrialization, urbanization, and growing populations (Khezri et al. 2021; Sadorsky 2014; Shahbaz et al. 2013). In general, agriculture, industry, fishing, and international commerce degrade water quality, alter the terrain, and have a detrimental effect on the atmosphere (Vitousek et al. 2018). According to Niewöhner et al. (2016), up to 50 percent of the land surface has been transformed by human activities, and CO_2_ emissions from human activities have steadily risen over the last several decades. Li et al. (2019) argue that economic activities must consume natural resources like minerals, forests, water, and land. Sharma et al. (2021) argue that economic activities exacerbate deforestation, exacerbate the use of pesticides, and amplify urbanization. They strengthen industrialization, mining, disappearing coastal areas, and building dams. They have all destroyed land, air, and water quality and the alteration of the ecological system. Economic expansion is a structural transformation that increases each country’s industrial activity and energy consumption. Therefore, growth is closely tied to energy consumption, which is closely tied to pollution levels. Grossman and Krueger (1991) introduced the EKC hypothesis, which asserts the economic growth-environmental degradation nexus and demonstrates that economic activities lead to an environmental deficit in the initial phase of economic growth. When a threshold of high economic growth is reached, the ecological destruction caused by economic activities subsides. The validity of this hypothesis has been assessed through various empirical studies. Some scholars have proved this hypothesis, and some have rejected it. However, all theorists agree on one crucial point: economic activity and human activities cause environmental damage (Majeed et al. 2021). In this context, Stern (1998) and Stokey (1998) proposed three channels via which economic development may affect environmental quality: (a) scale effects, (b) technique effects, and (c) composition impacts. These three criteria might influence the real environmental effects of economic development. In addition, the factor endowment hypothesis asserts that countries with a high level of capital endowments that make an effort to expand capital-intensive industries and absorb foreign direct investments will experience an increase in pollution-intensive industries, resulting in a decline in environmental efficiency (Zheng et al. 2015).

Usman et al. (2020) found that economic expansion has a detrimental influence on the ecological footprints of Africa and Europe. In addition, economic liberalization has been proven to have negative environmental repercussions in Africa and the United States. In addition, they noted that foreign direct investment and primary energy use contribute to Africa and Asia’s ecological footprints. Yang et al. (2021) analyzed the effects of industrialization, economic growth, and globalization on the ecological footprint. They determined that industrialization and economic expansion are largely responsible for pollution levels. Turnbull et al. (2018) and Clausen and York (2008) demonstrate that economic growth is connected with declines in aquatic biodiversity, agri-food system degradation, and an increase in the fishing footprint. Song et al. (2013), Clark and Longo (2019), and Solarin et al. (2021) provided empirical evidence of the detrimental consequences of economic activities on environmental efficiency.

### Energy use – environmental performance

Energy is one of the essential production components in theories of economic development and the direction of expansion, which hinders energy consumption that consequently plays a vital role in environment-economic growth literature (Bölük and Mert 2014; Omri and kahouli 2014). Theoretically, energy consumption and demand for fossil fuel increases at the initial level of economic growth (Destek and Sinha 2020). The usage of energy results in CO_2_ and SO_2_ emissions that affect environmental quality. Almost all economic sectors, including the transportation sector, agriculture, mining, industry, and service sector, are energy-intensive, and many developing countries have lax regulations against polluting activities, which transforms these countries into pollution havens and exacerbates ecological footprints (Danish 2018; Mamkhezri et al. 2022; Sarkodie and Strezov 2019). To preserve environmental quality, enterprises must implement less harmful technology and renewable energy as economic development and environmental consciousness rise.

Generally, the energy consumption- environmental quality nexus analysis is based on two main streams of theories. The first emphasizes efficiency improvements in energy consumption to reduce toxic pollutants and control environmental demolitions, hence cutting fossil fuel energy subsidies is proposed to control energy consumption (Shahzad et al. 2021). Other literature confirms that economic complexity, good and services production structures, and economy of scale, play a predominant role in the effect of energy consumption on ecological footprints (Danish 2018; Khezri et al. 2022; Neagu and Teodoru 2019; Ozcan et al. 2019; Ulucak and Lin 2017). The early investigation on the effect of energy consumption on ecological footprint revealed that non-renewable energy usage had negative effects on ecological footprint. However, Abbasi and Abbasi (2000) and Saidur et al. (2011) argue that all types of energy (renewable/non-renewable) lead to environmental impacts, and renewable energy is not the universal cure. In this regard, Saidi and Omri (2020) and Nathaniel and Iheonu (2019) suggest that burning biomass materials generated by using renewable energy pollutes water and land. According to Shahzad et al. (2021), the increased consumption of diverse energies such as oil, coal, gas, electricity, and water is the primary cause of carbon emissions and ecological footprint in the United States. The United States’ heavy reliance on fossil fuels threatens the environment, global warming, public health, etc (Ahmed et al. 2020; Alola et al. 2019; Destek and Sinha 2020; Karimi et al. 2022; Zafar et al. 2019).

Reviewing the tremendous literature that has been done about measuring environmental performance, the DEA models have been widely applied in environmental performance assessments. These models consider labor, capital, and energy consumption as the main inputs in countries’ economic activities, leading to gross domestic product and undesirable outputs as ecological damages in the production process. There is a large bulk of empirical literature using DEA models in assessing environmental performance in OECD countries, as Xue et al. (2022), Wang et al. (2022), Fidanoski et al. (2021), Sun et al. (Iram et al. 2020), Iram et al. (Iram et al. 2020), Gavurova et al. (2018), Simsek (2014), Hoang and Alauddin (2012) among others. Therefore, we shall describe the use of DEA models in assessing environmental performance below.

### Research Methodology

**Fig. 1:**
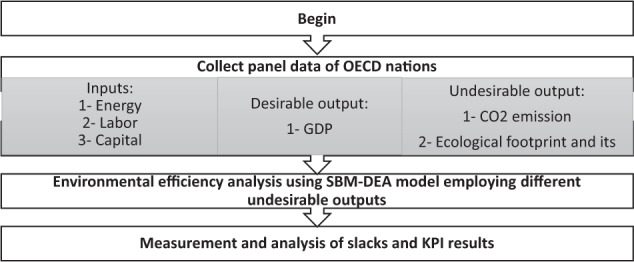
The flowchart ot the methodology

The two primary aims of this study are as follows: First, we aim to evaluate the environmental efficiency of OECD countries based on different undesirable outputs, including ecological footprint, detailed categories, and CO_2_ emissions. The Slack-Based Measurement DEA (SBM) model is utilized to accomplish this goal, which is thoroughly presented in sections Data Envelopment Analysis (DEA) and Slack-Based Measurement-DEA models. Second, we intend to decompose the environmental efficiency scores to analyze each country’s ecological performance more precisely. In section Key Performance Indicators (KPI), the method for measuring these KPIs is described. The flowchart depicted in Fig. 1 summarizes the methodology of this paper.

### Data Envelopment Analysis (DEA)

Data Envelopment Analysis (DEA) is a potent nonparametric technique for analyzing the relative performance of a group of comparable decision-making units (DMUs) with various inputs and outputs. Based on Kopp’s (1981) boundary notion, Charnes et al. (1978) introduced the CCR model as the first DEA framework. The CCR model assumes constant returns to scale (CRS) and assesses the DMUs’ technical efficiency. Banker et al. (1984) extended the CRS premise of the previous model. They created the BCC model, which assumes variable returns to scale (VRS) and assesses the pure technical efficiency of the DMUs.

Suppose a set of n DMUs denoted by (*j* = 1, .. ,*n*), and each DMU has *m* inputs and *s* outputs. *x*_*ij*_ and *y*_*rj*_ represent the i^th^ input and r^th^ output of DMU_j_, respectively. The CCR model can be formulated as a linear programming model represented in Eq. (1):

1 $$\begin{array}{l}min\,\theta _k\\ s.t.\,\mathop {\sum}\limits_{j = 1}^n {\lambda _jx_{ij} \le \theta _kx_{ik}\,i = 1, \ldots ,\,m} \\ \mathop {\sum}\limits_{j = 1}^n {\lambda _jy_{rj} \ge y_{rk}\,r = 1,\, \ldots ,\,s} \\ \lambda _j \ge 0\,\forall j\end{array}$$

Where the optimal value of the model (${\boldsymbol{\theta}}^*_{k}$) reflects the relative efficiency of the DMU, and it ranges between 0 and 1. The frontier’s efficient DMUs have a score of 1 for efficiency. Efficiency ratings of less than 1 show the gap between an inefficient DMU and an efficient frontier. *λ*_*j*_ represents the vector of weights assigned to inputs and outputs. The BCC model based on the variable return to scale (VRS) assumption can be formulated by adding a convexity constraint ($\mathop {\sum}\nolimits_{j = 1}^n {\lambda _j = 1}$) to the above CCR model.

### Slack-Based Measurement-DEA models

The basic DEA models illustrated in the previous section, CCR, and BCC, are known as radial models, which are not accurate enough in practice and suffer some drawbacks. First, radial models adjust all inputs or outputs by the same proportion to make DMUs efficient, while in the real world, inputs (or outputs) don’t necessarily behave in a proportional way (Muhammad et al. 2018). Second, they fail to consider the slack of indicators, and when there is a non-zero slack of inputs or outputs, the radial DEA models overestimate the efficiency of DMUs (Jiang et al. 2021). Tone et al. (2020) created a non-radial slack-based measurement DEA (SBM-DEA) model that dealt with slacks directly and disregarded the assumption of proportional changes in inputs and outputs to solve these disadvantages. Although Tone’s SBM-DEA model exhibited more discriminating power than conventional radial DEA models, it did not take into account undesirable outcomes. Chang et al. (2013) enhanced the SBM-DEA model for the first time by including undesirable output directly into the goal function and restrictions in Eq. (2):

2 $$\begin{array}{l}\theta ^ \ast = min\frac{{1 - \frac{1}{m}\mathop {\sum}\nolimits_{i = 1}^m {\frac{{s_i^ - }}{{x_{ik}}}} }}{{1 + \frac{1}{{q_1 + q_2}}\left( {\mathop {\sum}\nolimits_{r = 1}^{q_1} {\frac{{s_r^g}}{{y_{rk}^g}}} + \mathop {\sum}\nolimits_{r = 1}^{q_2} {\frac{{s_r^b}}{{y_{rk}^b}}} } \right)}}\\ s.t.\mathop {\sum}\limits_{j = 1}^n {\lambda _jx_{ij} + s_i^ - = x_{ik}\,i = 1,\, \ldots ,\,m} \\ \mathop {\sum}\limits_{j = 1}^n {\lambda _jy_{rj}^g - s_r^g = y_{rk}^g\,r = 1,\, \ldots ,\,q_1} \\ \mathop {\sum}\limits_{j = 1}^n {\lambda _jy_{rj}^b + s_r^b = y_{rk}^b\,r = 1,\, \ldots ,q_2} \\ \lambda _j,s_i^ - ,s_r^g,\,s_r^b \ge 0\,\forall i,j,r\end{array}$$

Where *x*_*ik*_, $y_{rk}^g$ and $y_{rk}^b$ depict inputs, desirable and undesirable outputs of DMU_k_. Slack variables are denoted by $s_i^ -$, $s_r^b$ and $s_r^g$, showing the excess in inputs and undesirable outputs, and shortfall in desirable outputs, respectively. The optimal value of the model, θ^*^, refers to the overall efficiency of the considered DMU, which is also called environmental efficiency and ranges between 0 to 1. A DMU is environmentally efficient if θ^*^ = 1, and all the slack variables are equal to zero. In contrast, an inefficient DMU has an efficiency score of less than one and at least one non-zero slack variable with a positive value. Inefficient DMUs may be improved by reducing the surplus of inputs and undesired outputs or increasing the lack of desirable outputs.

### Key Performance Indicators (KPI)

Energy efficiency and Carbon efficiency were two of the most widely used KPIs in the DEA context (Choi et al. 2012; Iftikhar et al. 2018; Park et al. 2016). Energy efficiency was first produced by Hu and Wang (2006) based on the “ratio of target to real” concept and later developed by Zhou et al. (2006) by incorporating undesirable outputs in energy efficiency analysis. We utilize Chang et al. (2013)‘s method suggested under the SBM-DEA model for calculating KPIs. This paper proposes ecological footprint efficiency as a substitute for carbon efficiency. Besides, five more KPI measures are developed based on the different ecological footprint categories to evaluate the carbon footprint efficiency, cropland efficiency, fishing grounds efficiency, forest area efficiency, and grazing land efficiency performances. The KPIs can be estimated as follows:

3 $$Energy\,Efficiency\,\left( {EE} \right) = \frac{{E_k - s_E^ - }}{{E_k}}$$

4 $$Ecological\,Footprint\,Efficiency\,\left( {EFE} \right) = \frac{{EF_k - s_{EF}^b}}{{EF_k}}$$

5 $$Carbon\,Footprint\,Efficiency\,\left( {CFE} \right) = \frac{{CF_k - s_{CF}^b}}{{CF_k}}$$

6 $$Cropland\,Efficiency\,\left( {EE} \right) = \frac{{CE_k - s_{CE}^b}}{{CE_k}}$$

7 $$Fishing\,Grounds\,Efficiency\,\left( {FGE} \right) = \frac{{FG_k - s_{FG}^b}}{{FG_k}}$$

8 $$Forest\,Area\,Efficiency\,\left( {FAE} \right) = \frac{{FA_k - s_{FA}^b}}{{FA_k}}$$

9 $$Grazing\,Land\,Efficiency\,\left( {GLE} \right) = \frac{{GL_k - s_{GL}^b}}{{GL_k}}$$

Where the actual values of energy (*E*_*k*_), ecological footprint (*EF*_*k*_), carbon footprint (*CF*_*k*_), cropland (*CE*_*k*_), fishing grounds (*FG*_*k*_), forest area (*FA*_*k*_), and grazing land (*GL*_*k*_) for *DMU*_*k*_ are deducted from corresponding slacks to measure the target value of each variable, and then KPIs are calculated through the ratio of the target to the actual values.

### Data Selection and Description

**Fig. 2:**
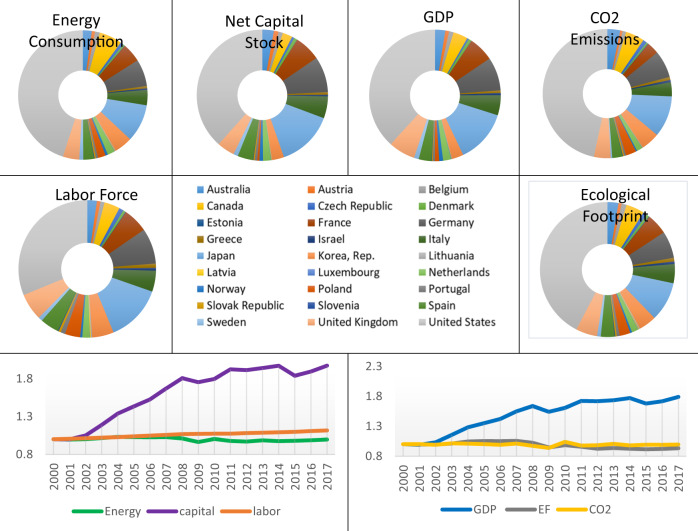
Research area and graphical representation of basic data

This research examines the environmental performance of 27 OECD nations (Fig. 2) using panel data gathered from 2000 to 2017 for a total of eighteen years. The SBM-DEA model is implemented with energy consumption, labor force, and net capital stock as inputs, Gross Domestic Product (GDP) as a desirable output, and CO_2_ emissions and ecological footprint (EF) (with its various categories including carbon footprint, cropland, fishing grounds, forest area, and grazing land) as undesirable outputs. As discussed in the introduction, the ecological footprint is a more comprehensive metric for evaluating the impact of human activities on the natural environment (Ahmad et al. 2020). It is a much better option than CO_2_ emissions for assessing environmental quality (Nathaniel and Khan 2020). In this respect, we evaluate and compare environmental efficiency ratings based on CO_2_ emissions and Ecological Footprint undesirable outputs. In addition, we utilize each Ecological Footprint category, such as carbon footprint, fishing grounds, cropland, forest area, and grazing land, as independent negative aspects to study the environmental performance of OECD countries in depth. The data on the EF is extracted from the databases of the global footprint network (www.footprintnetwork.org), and the rest data is retrieved from the OECD data (www.data.oecd.or). Table 1 presents the summary of descriptive statistics of the considered variables. Figure 2 illustrates the distribution and aggregate input and output data trends in the OECD countries from 2010 to 2017.

**Table 1:** Descriptive statistics of the input and output variables over the years 2000–2017

	Variable	unity	Minimum	Maximum	Mean	S.D.
Inputs	Energy Consumption	Thousand tons of oil equivalent	2582.98	1,576,083.72	125,154.90	285,431.88
	Labor force	Persons	188,742	164,326,552	18,447,738.5	30,942,581
	Net Capital Stock	Current US dollar	18,236.2	59,547,725	4,246,791.4	8,839,156.7
Desirable output	GDP	Current US dollar (thousands)	5,686,579	19,542,979,183	1,416,446,779	2,903,026,259
Undesirable output	CO_2_ emissions	Thousand tons	6930	5,776,410	426,952.7	1,013,204.3
	Ecological Footprint (EF)	Global hectares	6,303,881.5	3,054,065,304	242,497,566.5	521,401,699.4
	Ecological footprint components					
	Carbon footprint	Global Hectares	2,781,968.2	2,240,391,046.3	167,409,437.8	383,891,691.3
	Cropland		266,291.4	332,418,642.3	29,884,670.3	52,102,827
	Fishing grounds		858.3	63,855,230.5	7,024,290.5	11,746,520.5
	Forest area		292,533.7	375,691,500.4	25,165,934.5	57,221,967.1
	Grazing land		135.651	115,405,976.4	9,996,563.4	19,458,914.8

As shown in Fig. 2, the United States utilizes the highest proportion of inputs, including energy consumption, net capital stock, and labor force, with respective shares of 45 percent, 39 percent, and 31 percent. This country generates the highest proportion of both desirable and undesirable outputs, including GDP (38 percent), Ecological Footprint (42 percent), and CO_2_ emissions (47 percent). Japan ranks second on the input and output variables list, with an average share of 11 percent, after the United States. Luxembourg has the lowest labor force and Ecological Footprint among the countries with the lowest share. Estonia has the lowest net capital stock, energy consumption, and GDP among the countries with the lowest share.

Considering the aggregate time-series trends of variables in Fig. 2, while net capital stock and labor force rose by 96% and 11%, respectively, total energy consumption remained unchanged throughout the study period. GDP increased by almost 78% from 2000 to 2017 for OECD countries, but CO_2_ emissions and Ecological Footprint decreased somewhat.

Moreover, a Pearson correlation test has been performed to calculate the correlation coefficients among the input and output variables. The results are reported in Table 2. Considering the correlation matrix, net capital stock, energy consumption, and labor force significantly positively correlate with GDP and Ecological Footprint.

**Table 2:** Correlation matrix of input and output dataset

	Energy Consumption	Net Capital Stock	Labor Force	GDP	Ecological footprint	CO_2_
Energy Consumption	1					
Net Capital Stock	0.980	1				
Labor Force	0.967	0.988	1			
GDP	0.985	0.997	0.990	1		
Ecological footprint	0.999	0.984	0.974	0.989	1	
CO_2_	0.998	0.979	0.967	0.983	0.998	1

Particularly, there is a very strong correlation (0.99) between net capital stock and labor force with the output GDP that emphasizes the importance of net capital stock and labor force as inputs of the DEA model. In addition, energy consumption is highly correlated with Ecological Footprint, showing that the more energy is consumed, the more environmental impact is made. Consequently, the positive correlation between inputs and outputs meets the isotonicity property of the DEA model, which stipulates that as inputs increase, outputs tend to rise as well, making this formulation suitable for statistical efficiency analysis.

## Results and Discussion

### Environmental Efficiency Analysis

The SBM-DEA framework was applied to compute the relative efficiency scores represented in Table 3 for OECD countries from 2000 to 2017. According to the results obtained, Lithuania has the lowest efficiency score at 0.210; on the contrary, Luxembourg, Norway, and the United Kingdom reach the maximum efficiency score of 1.000. Besides, five countries, Denmark, Poland, Canada, Israel, and Japan, reach an average efficiency score greater than 0.7, and there are three countries, including Estonia, the Czech Republic, and Slovak, with an average efficiency score of less than 0.3. The other 17 countries’ efficiency scores are located between 0.3 and 0.7. Overall, the average efficiency score is 0.593, showing the existing improvement potential of at least 0.4 for OECD countries.

**Table 3:** Relative efficiency scores for OECD countries, 2000–2017

	2000	2001	2002	2003	2004	2005	2006	2007	2008	2009	2010	2011	2012	2013	2014	2015	2016	2017	average
Australia	0.484	0.477	0.448	0.455	0.491	0.471	0.455	0.470	0.492	0.529	0.585	0.580	0.622	0.625	0.624	0.742	0.628	0.638	0.545
Austria	0.577	0.625	0.636	0.648	0.652	0.563	0.538	0.569	0.547	0.608	0.550	0.509	0.482	0.489	0.533	0.593	0.586	0.589	0.572
Belgium	0.561	0.608	0.606	0.624	0.631	0.555	0.537	0.564	0.527	0.600	0.560	0.531	0.498	0.509	0.553	0.581	0.575	0.581	0.567
Canada	1	1	1	1	1	1	1	1	0.809	0.763	0.723	0.744	0.704	0.638	0.547	0.529	0.493	0.515	0.804
Czech	0.198	0.215	0.232	0.243	0.243	0.268	0.276	0.299	0.308	0.332	0.317	0.305	0.290	0.294	0.297	0.308	0.309	0.325	0.281
Denmark	0.666	0.735	1	1	1	1	1	1	1	1	1	0.677	0.652	1	1	1	1	1	0.929
Estonia	0.212	0.219	0.234	0.252	0.256	0.282	0.297	0.321	0.326	0.317	0.319	0.335	0.329	0.334	0.338	0.329	0.336	0.355	0.299
France	0.652	0.709	0.707	0.746	0.733	0.633	0.610	0.634	0.603	0.667	0.621	0.573	0.528	0.545	0.595	0.662	0.677	0.676	0.643
Germany	0.594	0.659	0.673	0.698	0.695	0.592	0.562	0.600	0.562	0.621	0.578	0.552	0.516	0.532	0.593	0.665	0.699	0.718	0.617
Greece	0.436	0.472	0.481	0.531	0.543	0.515	0.506	0.528	0.521	0.578	0.527	0.445	0.408	0.422	0.441	0.416	0.423	0.436	0.479
Israel	0.826	0.833	0.686	0.625	0.549	0.565	0.552	0.563	0.651	0.797	0.794	0.763	0.729	0.831	0.812	0.774	1	1	0.742
Italy	0.625	0.692	0.696	0.721	0.713	0.609	0.584	0.604	0.575	0.635	0.579	0.534	0.493	0.510	0.547	0.595	0.597	0.604	0.606
Japan	1	1	1	1	1	0.658	0.582	0.538	0.536	0.662	0.678	0.610	0.608	0.520	0.526	0.616	0.719	0.659	0.717
Korea	0.409	0.399	0.403	0.391	0.376	0.401	0.395	0.391	0.330	0.347	0.373	0.346	0.343	0.356	0.372	0.408	0.392	0.402	0.380
Latvia	0.223	0.241	0.250	0.278	0.269	0.300	0.308	0.327	0.373	0.368	0.366	0.364	0.361	0.381	0.376	0.364	0.359	0.382	0.327
Lithuania	0.141	0.153	0.156	0.164	0.178	0.190	0.199	0.229	0.247	0.250	0.226	0.230	0.224	0.239	0.235	0.231	0.246	0.252	0.211
Luxembourg	1	1	1	1	1	1	1	1	1	1	1	1	1	1	1	1	1	1	1.000
Netherlands	0.616	0.677	0.668	0.688	0.687	0.598	0.584	0.619	0.601	0.679	0.594	0.556	0.509	0.541	0.595	0.676	0.714	0.728	0.629
Norway	1	1	1	1	1	1	1	1	1	1	1	1	1	1	1	1	1	1	1.000
Poland	0.367	0.418	0.449	0.453	1	1	1	1	1	1	1	1	1	1	1	1	1	1	0.871
Portugal	0.399	0.419	0.412	0.444	0.427	0.410	0.403	0.417	0.402	0.447	0.421	0.388	0.370	0.391	0.413	0.405	0.432	0.435	0.413
Slovak	0.175	0.184	0.193	0.226	0.234	0.248	0.258	0.280	0.273	0.300	0.294	0.285	0.287	0.290	0.317	0.311	0.309	0.304	0.265
Slovenia	0.336	0.357	0.366	0.398	0.378	0.372	0.366	0.400	0.395	0.437	0.398	0.371	0.346	0.357	0.389	0.383	0.381	0.401	0.379
Spain	0.457	0.507	0.499	0.532	0.519	0.474	0.468	0.487	0.482	0.549	0.509	0.465	0.441	0.456	0.492	0.516	0.532	0.547	0.496
Sweden	0.730	0.722	0.687	0.741	0.774	0.612	0.673	0.659	0.619	0.643	0.635	0.620	0.589	0.619	0.640	0.735	0.721	0.727	0.675
U.K.	1	1	1	1	1	1	1	1	1	1	1	1	1	1	1	1	1	1	1.000
U.S.A	0.682	0.768	0.696	0.635	0.583	0.522	0.501	0.486	0.450	0.532	0.516	0.473	0.485	0.488	0.525	0.639	0.617	0.630	0.568

**Fig. 3:**
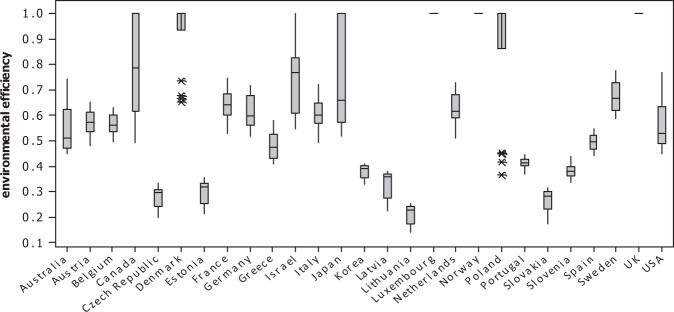
Relative efficiency scores’ boxplot for OECD countries over the study period

Moreover, the dispersion of the efficiency scores for each country is presented in the boxplot of Fig. 3, according to which Poland has the most scattered efficiency scores. On the contrary, Portugal is considered the most concentrated nation among inefficient countries. As mentioned in the section (introduction), CO_2_ emissions were the most common variable used in previous studies to reflect environmental effects in DEA models. To explore the difference, we compare the results of the previous section with those obtained from an SBM-DEA model in which CO_2_ emissions are considered undesirable output. Besides, since ecological footprint itself is divided into different categories of carbon footprint, cropland, fishing grounds, forest area, and grazing land, we entered these elements into the SBM-DEA model separately to analyze the impact of each category on the OECDs’ environmental efficiency scores.

Table 4 Summarizes the average efficiency scores obtained from SBM-DEA models based on different undesirable outputs. Considering CO_2_ emissions and ecological footprint independently as undesirable outputs, we obtained different values for the environmental performance of OECD nations. CO_2_ emissions and ecological footprint overlap, but since CO_2_ emission is the most widely used variable in the relevant literature, a comparison between these two variables has been conducted to appear the difference and similarities results with prior studies. The results revealed that the environmental efficiency scores based on CO_2_ emissions are close to those based on the ecological footprint. This is quite reasonable because OECD countries’ ecological footprint and CO_2_ emissions are highly correlated (Table 2). Ecological footprint-oriented results are more reliable since the corresponding index represents the environmental quality far better than the extensively used indicator, CO_2_ emissions.

**Table 4:** Average environmental efficiency based on different undesirable outputs for OECD countries, 2000–2017

Country	Average environmental efficiency based on…						
	Ecological Footprint	CO_2_ emissions	Carbon footprint	cropland	Fishing grounds	Forest area	Grazing land
Australia	0.545	0.497	0.527	0.575	0.590	0.522	0.559
Austria	0.572	0.566	0.536	0.548	0.879	0.546	0.593
Belgium	0.567	0.559	0.549	0.521	0.557	0.568	0.532
Canada	0.804	0.764	0.807	0.785	0.751	0.778	0.802
Czech	0.281	0.272	0.281	0.275	0.331	0.271	0.283
Denmark	0.929	0.942	0.934	0.921	0.959	0.923	0.920
Estonia	0.299	0.293	0.308	0.305	0.472	0.282	0.345
France	0.643	0.682	0.610	0.579	0.597	0.617	0.615
Germany	0.617	0.563	0.570	0.563	0.945	0.617	0.695
Greece	0.479	0.469	0.476	0.446	0.487	0.540	0.458
Israel	0.742	0.724	0.731	0.705	0.779	0.865	0.774
Italy	0.606	0.601	0.579	0.557	0.614	0.610	0.559
Japan	0.717	0.648	0.661	0.735	0.626	0.983	0.985
Korea	0.380	0.362	0.373	0.375	0.329	0.514	0.463
Latvia	0.327	0.369	0.368	0.312	0.304	0.296	0.363
Lithuania	0.211	0.219	0.215	0.205	0.203	0.204	0.215
Luxembourg	1	1	1	1	1	1	1
Netherlands	0.629	0.586	0.589	0.561	0.676	0.823	0.556
Norway	1	1	1	0.971	0.917	0.982	0.981
Poland	0.871	0.871	0.872	0.868	0.884	0.871	0.972
Portugal	0.413	0.419	0.412	0.386	0.367	0.599	0.400
Slovak	0.265	0.261	0.263	0.261	0.298	0.255	0.309
Slovenia	0.379	0.373	0.374	0.368	0.424	0.364	0.415
Spain	0.496	0.492	0.486	0.449	0.440	0.591	0.528
Sweden	0.675	0.896	0.649	0.628	0.649	0.585	0.622
U.K.	1	1	1	1	1	0.991	1
U.S.A	0.568	0.528	0.538	0.565	0.560	0.555	0.604
average	0.593	0.591	0.582	0.573	0.616	0.620	0.613

Table 4 also shows the environmental efficiency resulting from different ecological footprint categories. Luxembourg is the only country that operates at the most optimum level (p^*^ = 1) in all categories. Among OECD nations, Luxembourg had the highest GDP per capita and ranked 8^th^ out of 27 on average in ecological footprint categories per GDP. In contrast, Lithuania was the least environmentally efficient country for all the models calculated based on carbon footprint, cropland, fishing grounds, forest area, and grazing land, with average efficiency scores of 0.215, 0.205, 0.203, 0.204, and 0.215, respectively. In more detail, Lithuania’s per capita GDP is lower than all of the OECD nations except Estonia. At the same time, it has the highest consumption of ecological resources and leaves the most carbon footprint per GDP. Therefore, such a combination of environmental and economic variables turns Lithuania into the country with the lowest efficiency scores in all the models. Like Luxembourg and Lithuania, we can compare the environmental efficiency of other OECD nations obtained from different ecological footprint categories, leading to a thorough and valuable analysis.

In addition, the assessment of environmental efficiency based on different ecological footprint categories provides the opportunity to identify each category’s impact on each country’s environmental efficiency. For example, Austria reaches the relative efficiency scores of 0.536, 0.548, 0.879, 0.546, and 0.593 from the perspectives of carbon footprint, cropland, fishing grounds, forest area, and grazing land. Comparing these scores illustrated that the fishing grounds’ current consumption as an ecological resource poses the least threat and danger to Austria’s environmental efficiency. In contrast, the current consumptions of other resources have relatively greater impacts on its environmental efficiency. Overall, the average environmental efficiency for all OECD nations based on ecological footprint or its detailed categories is around 0.6, which leaves room for improvement by 0.4.

### Analysis of slacks and KPI results

**Fig. 4:**
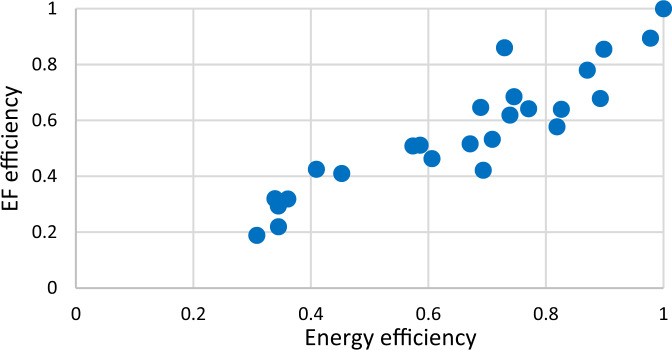
Scatter plot of energy efficiency and ecological footprint efficiency

**Fig. 5:**
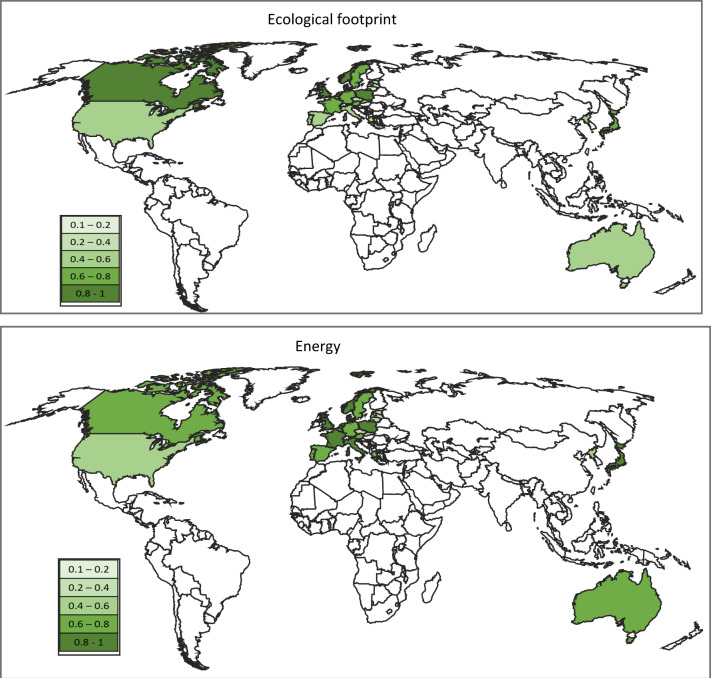
The ecological footprint and energy efficiency spatial distribution map of OECD countries from 2000 to 2017

In addition to energy efficiency (EE), we have proposed six new key performance indicators (KPI), including ecological footprint efficiency (EFE), carbon footprint efficiency (CFE), cropland efficiency (CE), fishing grounds efficiency (FGE), forest area efficiency (FEE) and grazing land efficiency (GLE). Table 5 presents the obtained results for all KPIs. In terms of energy efficiency (EE), three countries, including Luxembourg, Norway, and United Kingdom, are energy efficient. Among energy-inefficient countries, Denmark reaches the highest energy efficiency of 0.978, and Slovak, Estonia, Czech Republic, Latvia, and Lithuania reach the lowest scores of 0.360, 0.345, 0.344, 0.338, and 0.307, respectively. Moreover, the ecological footprint efficiency of Denmark and Lithuania are 0.894 and 0.188, ranking first and last among inefficient countries, analogous to energy efficiency results. The scatter plot and spatial distribution map in Figs. 4 and 5 corroborate that there is consistency between ecological footprint efficiency and energy efficiency. Energy-inefficient countries expose their ecology to a greater risk level. Previous studies have extensively shown such consistency between energy efficiency and CO_2_ emissions efficiency (Demiral and Sağlam 2021; Park et al. 2016).

**Table 5:** Average KPIs for OECD countries, 2000–2017

Country	Energy efficiency	Ecological footprint efficiency	Carbon footprint efficiency	Cropland efficiency	Fishing grounds efficiency	Forest area efficiency	Grazing land efficiency
Australia	0.670	0.516	0.322	0.604	0.524	0.440	0.376
Austria	0.738	0.619	0.392	0.533	1	0.525	0.632
Belgium	0.585	0.511	0.393	0.384	0.540	0.519	0.385
Canada	0.729	0.860	0.894	0.793	0.709	0.749	0.901
Czech	0.344	0.293	0.251	0.250	0.620	0.191	0.314
Denmark	0.977	0.894	0.906	0.863	0.911	0.866	0.857
Estonia	0.345	0.219	0.251	0.280	0.510	0.074	0.502
France	0.826	0.639	0.446	0.441	0.389	0.577	0.515
Germany	0.770	0.641	0.424	0.484	0.996	0.680	0.816
Greece	0.693	0.421	0.344	0.232	0.394	0.660	0.298
Israel	0.892	0.678	0.609	0.551	0.749	0.891	0.767
Italy	0.818	0.577	0.387	0.408	0.506	0.592	0.385
Japan	0.870	0.779	0.553	0.863	0.369	0.997	0.984
Korea	0.409	0.425	0.342	0.423	0.083	0.834	0.719
Latvia	0.338	0.319	0.498	0.254	0.186	0.098	0.518
Lithuania	0.307	0.188	0.212	0.101	0.051	0.077	0.232
Luxembourg	1	1	1	1	1	1	1
Netherlands	0.688	0.646	0.442	0.453	0.670	0.914	0.392
Norway	1	1	1	0.968	0.803	0.987	0.969
Poland	0.898	0.855	0.850	0.821	0.912	0.845	0.996
Portugal	0.605	0.463	0.347	0.286	0.111	0.788	0.343
Slovak	0.360	0.318	0.288	0.289	0.573	0.235	0.530
Slovenia	0.452	0.410	0.361	0.356	0.600	0.324	0.476
Spain	0.708	0.532	0.357	0.3	0.150	0.736	0.556
Sweden	0.745	0.684	0.531	0.583	0.581	0.392	0.521
U.K.	1	1	1	1	1	0.982	1
U.S.A	0.573	0.508	0.309	0.589	0.572	0.490	0.592
Average	0.679	0.592	0.508	0.522	0.574	0.610	0.614

Table 5 also shows KPIs representing different ecological footprint categories, including carbon footprint, cropland, fishing grounds, forest area, and grazing land. These detailed KPIs allow one to scrutinize each country’s ecological performance more precisely. Luxembourg is the most efficient country among OECD nations as it operates all ecological KPIs at the most optimum level. In the following, we explore the KPIs of some countries in detail. For example, Austria reaches the optimum score for fishing grounds efficiency; however, its other ecological KPIs are around 0.5. The basic data explored that Austria ranks 5^th^ in fishing grounds consumption and ranks 21st, 9th,16th, and 11th among the other 27 listed countries in terms of carbon footprint, cropland, forest, and grazing land consumption. Therefore, utilizing a much lower amount of fishing grounds rather than other ecological resources results in greater fishing grounds efficiency for Austria. On the contrary, considering Korea as another example, it can be argued that its worst ecological performance relates to fishing grounds efficiency because Korea is among the countries with the highest fishing grounds per capita usage, ranking 25 out of 27 countries. According to the last row of Table 5, OECD nations’ ecological inefficiency could be mostly traced back to carbon footprint and cropland with the KPIs of 0.508 and 0.522, respectively. Therefore, the proposed KPIs in this paper have enough potential to provide valuable information about countries’ performance in detailed ecological footprint categories.

**Fig. 6:**
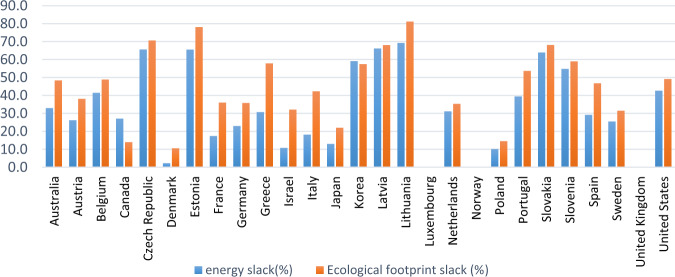
Graphical representation of average energy and ecological footprint slacks (%) for OECD countries

As the results of relative efficiency scores indicate, most of the OECD nations perform environmentally inefficiently, concluding that their input and output variables are located at a distance from the efficient frontier. The purpose of employing the SBM-DEA model in this paper is to determine these slacks and analyze excess inputs and undesirable outputs. Consequently, inefficient nations can accurately assess their status, develop appropriate measures to reduce slacks, and get closer to the efficient frontier. The estimated slack values with their associated percentage are represented in Table 6, and Fig. 6 shows the graphical representation of the energy and ecological footprint slacks for OECD countries from 2000 to 2017. Comparing these slacks with the KPI scores in Table 5, it can be concluded that low-ranked inefficient countries have high slacks in both energy input and ecological footprint output. As the performance score increases, the slack values converge to zero. For example, environmentally efficient countries, including Luxembourg, Norway, and United Kingdom, have zero slacks for both energy and ecological footprint. Lithuania, with the lowest environmental efficiency score, shows the highest energy and ecological footprint reduction potential of 69.2 and 81.1%, respectively. On the contrary, Denmark, which shows the highest environmental performance among inefficient countries, requires the lowest energy and ecological footprint reduction by 2.2 and 10.5%.

**Table 6:** Average slack variables for OECD countries, 2000 to 2017

Country	Energy slack (Mtoe)	Ecological footprint slack (10^6^ hectares*)	Carbon footprint slack (10^6^ hectares)	Cropland slack (10^6^ hectares)	Fishing grounds slack (10^6^ hectares)	Forest area slack (10^6^ hectares)	Grazing land slack (10^6^ hectares)
Australia	24.98 (32.9%)	83.30 (48.4%)	74.75 (67.4%)	7.92 (44.6%)	1.35 (47.7%)	12.86 (56.7%)	12.81 (75.7%)
Austria	6.96 (26.1%)	19.31 (38.1%)	20.91 (60.9%)	3.08 (46.9%)	0.00	2.96 (47.3%)	0.72 (36.2%)
Belgium	17.04 (41.4%)	37.73 (48.9%)	29.97 (61.0%)	7.37 (61.9%)	0.53 (47.5%)	4.12 (51.1%)	3.23 (62.6%)
Canada	52.25 (27.1%)	40.02 (14.0%)	20.17 (10.8%)	7.57 (21.3%)	1.42 (26.8%)	10.91 (25.0%)	1.19 (9.7%)
Czech	17.45 (65.6%)	43.26 (70.6%)	29.63 (75.2%)	5.94 (75.5%)	0.14 (38.2%)	7.54 (80.9%)	1.98 (69.3%)
Denmark	0.319 (2.2%)	4.44 (10.5%)	2.04 (9.3%)	0.94 (14.2%)	0.46 (11.6%)	0.83 (14.0%)	0.34 (13.2%)
Estonia	1.92 (65.5%)	7.46 (78.1%)	3.70 (75.3%)	0.65 (75.2%)	0.05 (68.3%)	3.20 (92.9%)	0.10 (51.7%)
France	27.92 (17.4%)	119.03 (36.0%)	106.94 (55.3%)	32.26 (56.2%)	7.70 (61.5%)	16.95 (43.3%)	9.04 (49.2%)
Germany	52.31 (23.0%)	151.54 (35.8%)	165.94 (57.7%)	34.01 (51.7%)	0.01 (0.3%)	13.83 (32.9%)	2.37 (18.2%)
Greece	5.79 (30.7%)	32.96 (57.8%)	23.90 (66.9%)	9.07 (76.4%)	0.69 (60.0%)	1.22 (35.0%)	2.96 (70.9%)
Israel	1.44 (10.8%)	13.21 (32.1%)	11.31 (38.8%)	2.89 (44.8%)	0.15 (25.1%)	0.32 (12.2%)	0.43 (22.7%)
Italy	23.54 (18.1%)	127.23 (42.3%)	115.95 (61.4%)	29.89 (58.8%)	3.74 (50.4%)	12.12 (41.1%)	13.60 (62.8%)
Japan	41.28 (13.0%)	136.38 (22.0%)	202.20 (44.3%)	7.69 (13.1%)	29.04 (59.6%)	0.10 (0.3%)	0.20 (1.4%)
Korea	90.25 (59.1%)	159.41 (57.5%)	133.55 (65.7%)	17.59 (58.1%)	21.38 (91.7%)	2.00 (17.5%)	1.75 (28.0%)
Latvia	3.66 (66.1%)	7.44 (68.0%)	1.89 (51.3%)	1.21 (74.9%)	0.29 (83.7%)	4.41 (90.4%)	0.18 (51.2%)
Lithuania	2.70 (69.2%)	12.10 (81.1%)	5.55 (78.5%)	2.60 (90.2%)	0.84 (95.1%)	3.00 (92.2%)	0.45 (78.6%)
Luxembourg	0.00	0.00	0.00	0.00	0.00	0.00	0.00
Netherlands	18.85 (31.1%)	35.84 (35.3%)	38.31 (57.0%)	9.18 (54.7%)	0.71 (40.2%)	0.62 (9.7%)	4.80 (60.6%)
Norway	0.00	0.00	0.00	0.13 (3.2%)	1.78 (23.5%)	0.04 (1.2%)	0.05 (3.0%)
Poland	6.58 (10.2%)	24.48 (14.5%)	14.88 (14.2%)	5.99 (20.0%)	0.16 (7.4%)	3.23 (11.8%)	0.01 (0.5%)
Portugal	7.28 (39.4%)	24.03 (53.7%)	18.13 (65.7%)	5.92 (71.2%)	3.14 (88.9%)	0.68 (29.4%)	1.86 (66.1%)
Slovak	7.08 (63.9%)	16.17 (68.1%)	11.35 (72.0%)	2.30 (72.6%)	0.09 (43.2%)	2.73 (77.2%)	0.26 (47.1%)
Slovenia	2.74 (54.8%)	6.04 (59.0%)	4.54 (64.7%)	0.86 (64.3%)	0.04 (40.0%)	0.97 (68.4%)	0.17 (52.8%)
Spain	26.31 (29.1%)	101.09 (46.8%)	85.17 (65.1%)	31.25 (70.0%)	13.86 (85.0%)	4.77 (31.7%)	3.43 (44.7%)
Sweden	8.62 (25.4%)	18.65 (31.5%)	14.99 (46.9%)	3.35 (41.7%)	0.55 (42.7%)	8.50 (65.2%)	1.80 (47.8%)
U.K.	0.00	0.00	0.00	0.00	0.00	0.52 (1.6%)	0.00
U.S.A	648.62 (42.7%)	1363.18 (69.6%)	1420.32 (69.6%)	114.57 (42.1%)	15.92 (43.1%)	159.35 (52.6%)	42.10 (41.0%)
Sum	1096.00	2584.30	2556.09	344.24	104.08	277.76	105.81
Average	40.59 (32.3%)	95.71 (40.7%)	94.67 (49.4%)	12.74 (48.3%)	3.854 (43.8%)	10.287 (40.1%)	3.919 (39.4%)

10^6^ hectare = Million hectares = MH

Table 6 also represents the detailed slacks for different categories of ecological footprint, namely carbon footprint, cropland, fishing grounds, forest area, and grazing land. It is worth noting that being aware of detailed potential reduction of each category could be very helpful for governments in drawing up future schemes and management practices. From the carbon footprint perspective, Luxembourg, Norway, and United Kingdom have zero redundancy. Denmark, Canada, and Poland show the lowest amount of carbon footprint excess among inefficient countries with 9.30, 10.79, and 14.17%, equivalent to 2.04, 20.17, and 14.88 million hectares, respectively. On the contrary, Lithuania has the highest reduction potential with 78.48% (equivalent to 5.54 MH), followed by Estonia with 75.33% (3.69 MH). The Czech Republic has 75.17% (or 29.62 MH). 24 countries out of 27 show an excessive carbon footprint with an average of 49.4%, indicating that inefficient countries must reduce their carbon footprint by more than 50% to achieve environmental efficiency.

Regarding other ecological footprint categories, Luxembourg is the only country with zero slacks, and Lithuania is again located at the farthest point from the efficient frontier. It must reduce the consumption of cropland, fishing grounds, forest area, and grazing land by 90.2, 95.1, 92.2, and 78.6%, respectively. Among non-zero slack nations, Norway, Germany, Japan, and Poland possess the smallest cropland slack (3.20%), fishing grounds slack (0.3%), forest area slack (0.3%), and grazing land slack (0.53%), respectively.

**Fig. 7:**
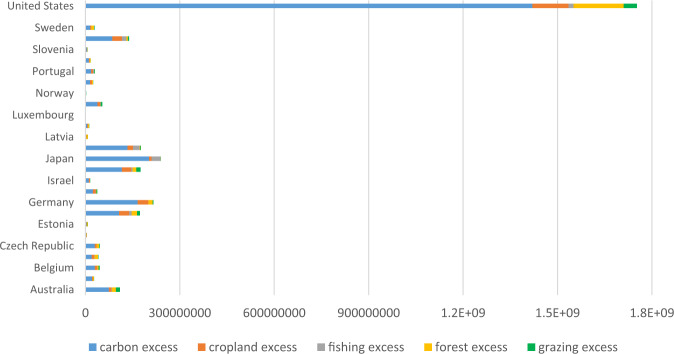
Graphical representation of detailed ecological footprint slacks for OECD countries, 2000–2017

For the whole OECD nations, carbon footprint, cropland, forest area, grazing land, and fishing grounds have the redundancy of 2556.09, 344.24, 277.76, 105.81, and 104.08 million hectares, respectively. This means that OECDs’ ecological inefficiency can be attributed largely to the presence of carbon footprint and cropland slacks and, afterward, to the forest area, grazing land, and fishing grounds. Figure 7 shows the graphical representation of each category’s slack ratio to the whole for OECD countries. These arguments lead to the conclusion that the industrial sector with a larger carbon footprint, and the agricultural sector as the main consumer of croplands, are mostly responsible for ecological inefficiency in our listed countries.

## Conclusion

In this study, an SBM-DEA model was applied to calculate the environmental efficiency of 27 OECD countries from 2000 to 2017 by using three inputs (labor force, energy consumption, and net capital stock), a single desirable output (GDP), and different undesirable outputs (CO_2_ emissions, ecological footprint with its detailed categories including carbon footprint, fishing grounds, cropland, forest area, and grazing land). Furthermore, energy efficiency and new proposed KPIs (ecological footprint efficiency, carbon footprint efficiency, cropland efficiency, fishing grounds efficiency, forest area efficiency, and grazing land efficiency) have been measured to scrutinize each country’s ecological performance more precisely. In addition, to explore excess inputs and undesirable outputs, the slack values of each variable were calculated and analyzed thoroughly.

The correlation coefficient among all input and output variables (higher than 96%) confirmed the use of DEA analysis in our data framework. The 0.99 correlation coefficient between energy consumption and ecological footprint indicates that energy use leads to a higher ecological footprint in OECD countries. Considering only ecological footprint as undesirable output, the results of SBM-DEA estimation showed that the average relative environmental efficiency for OECD countries is 0.59, and they have continuously slightly improved environmental efficiency from a mean of 0.56 in 2000 to 0.62 in 2017. Gavurova et al. (2018) applied the non-radial and non-oriented DEA approach (SBM model) with undesirable output under the condition of constant return to scale and concluded that the environmental performance of OECD nations improved between 1995 and 2014, which is confirmed by our estimation result. Luxemburg, the United Kingdom, and Norway reached the maximum efficiency score of 1.000 from 2000 to 2017, and this result is consistent with Kiani Mavi et al. (2022) who have used a combined method consisting of dynamic DEA analysis with goal programming and Li et al. (2019) who have applied Slack-based Metafrontier Dynamic DEA (SBM MFD-DEA) model. Lithuania, Slovak, Czech, and Estonia recorded minimum scores of environmental efficiencies. These results are in line with Mavi and Mavi (2019) who applied the ideal point method in combination with the dynamic DEA model and Gavurova et al. (2018), showing that the Eastern part of Europe has weak performance in environmental efficiency. To do sensitivity analysis, we also estimate the model considering only CO_2_ emission as undesirable output, resulting in different but close values for the environmental performance of OECD nations. Assessing the environment efficiency based on different ecological footprint categories showed that Luxemburg performs the best in all ecological footprint elements, and Lithuania recorded the worst environmental efficiency in all ecological footprint categories.

Luxemburg which reached the maximum efficiency score among OECD countries has set ambitious environmental objectives by 2030. Despite the rapid growth of GDP and the population, pressures on the environment have diminished significantly because it has a comprehensive set of laws and regulations to reduce pollutants emissions, manage waste, and protect nature. The U.K. as the second efficient country takes a leadership role in green innovation and it is very active in the promotion of eco-innovation, the circular economy, and new business models with environmental benefits. Such analysis reveals other involved factors influencing the environmental efficiency of OECD countries and can be comprehensively carried out in future studies.

Energy efficiency analysis and KPI results show that three countries, including Luxembourg, Norway, and United Kingdom, were energy efficient. At the same time, the Czech Republic, Latvia, and Lithuania reached the lowest energy efficiency scores. This result is according to the results of Simsek (2014) obtained from a bad output SBM-DEA model. In addition, the KPIs showed that energy-inefficient countries exposed their ecology to a greater risk level, confirmed by Park et al. (2016) using a non-radial slack-based DEA model and Demiral and Sağlam (2021) using both radial and non-radial DEA models. According to the detailed ecological KPIs analysis, Luxembourg, the United Kingdom, and Norway have recorded the top efficiency score in all ecological footprint components. OECD countries have the worst performance in carbon footprint efficiency and cropland efficiency.

In contrast, these countries perform best in energy efficiency. The remarkable point is that the fishing ground efficiency in Lithuania, South Korea, Portugal, and Spain is critical and lower than 15%. In addition, Estonia, Latvia, Lithuania, and Czech status in forest area efficiency are very acute and lower than 0.15.

Moreover, the detailed slacks for different categories of ecological footprint, namely carbon footprint, fishing grounds, cropland, forest area, and grazing land, indicate that Luxemburg is the only OECD country with zero deficit in all ecological footprint categories. Also, Lithuania is again located at the farthest point from the efficient frontier. However, the higher ecological footprint deficits belong to Lithuania (81.1%), Estonia (78.1%), Czech (70.6%), and the USA (69.6%). For the whole OECD countries, carbon footprint, cropland, forest area, grazing land, and fishing grounds had the redundancy of 2556.09, 344.24, 277.76, 105.81, and 104.08 million hectares, respectively. Therefore, OECDs’ ecological inefficiency could be largely attributed to the presence of carbon footprint and cropland slacks.

The concluding outcomes of this paper illustrate that Norway, Luxemburg, and United Kingdom are the most environmentally efficient countries in the period 2000–2017 in terms of environmental and ecological footprint efficiency. On the other hand, the lowest environmental and ecological footprint efficiencies were in countries like Lithuania, Slovak, the Czech Republic, Estonia, and the USA. In addition, OECD countries have experienced a low environmental efficiency improvement in this period. Moreover, Canada and Japan have low carbon footprint and cropland efficiency performance. These countries have main slacks in carbon footprint and cropland areas. In more detail, Lithuania, South Korea, Portugal, and Spain have critical status in fishing ground efficiency, and the forest area efficiency is very acute in Estonia, Latvia, Lithuania, and Czech.

The results of this paper could provide further insight into the investigated environmental efficiency measurements. They offer a wide view for policymakers in the decision-making process to identify ecological footprint components’ efficiency separately and prioritize the risky component of ecological footprints to make conservative policies. In future work, different methods of DEA can be used considering ecological footprint as the undesirable output to prevent a systematic error that may be caused by applying one single method.
